# Transcriptome profiling of ‘Kyoho’ grape at different stages of berry development following 5-azaC treatment

**DOI:** 10.1186/s12864-019-6204-1

**Published:** 2019-11-08

**Authors:** Da-Long Guo, Qiong Li, Xiao-Ru Ji, Zhen-Guang Wang, Yi-He Yu

**Affiliations:** 1 College of Forestry, Henan University of Science and Technology, Luoyang, 471023 Henan Province China; 2 Henan Engineering Technology Research Center of Quality Regulation and Controlling of Horticultural Plants, Luoyang, 471023 Henan Province China

**Keywords:** Kyoho, Grape, Ripening, Transcriptome, 5-azaC, DEG

## Abstract

**Background:**

5-Azacytidine (5-azaC) promotes the development of ‘Kyoho’ grape berry but the associated changes in gene expression have not been reported. In this study, we performed transcriptome analysis of grape berry at five developmental stages after 5-azaC treatment to elucidate the gene expression networks controlling berry ripening.

**Results:**

The expression patterns of most genes across the time series were similar between the 5-azaC treatment and control groups. The number of differentially expressed genes (DEGs) at a given developmental stage ranged from 9 (A3_C3) to 690 (A5_C5). The results indicated that 5-azaC treatment had not very great influences on the expressions of most genes. Functional annotation of the DEGs revealed that they were mainly related to fruit softening, photosynthesis, protein phosphorylation, and heat stress. Eight modules showed high correlation with specific developmental stages and hub genes such as *PEROXIDASE 4*, *CAFFEIC ACID 3-O-METHYLTRANSFERASE 1*, and *HISTONE-LYSINE N-METHYLTRANSFERASE EZA1* were identified by weighted gene correlation network analysis.

**Conclusions:**

5-AzaC treatment alters the transcriptional profile of grape berry at different stages of development, which may involve changes in DNA methylation.

## Background

Grape (*Vitis vinifera* L.) is one of the most important perennial woody fruit crops in the world. The grape berry is consumed whole or in the form of raisins or wine and has high nutritional, medicinal, and economic value [1], making it one of the most popular fruits. Grape berry exhibits change in pigmentation, sugar and organic acid contents, and other quality components during development and ripening [2] and is a useful model for studying fruit development.

Transcriptome sequencing is the main technology for investigating genome-wide changes in gene expression patterns, and has been used to study metabolic pathways and gene expression during fruit development in many plants. Most of the research has focused on climacteric fruits such as bayberry [3], pear [4, 5], kiwifruit [6], peach [7], tomato [8], and apricot [9], although recent studies have also investigated non-climacteric fruits such as sweet orange [10] and strawberry [11]. For example, cell wall biosynthesis, carbohydrate metabolism, the tricarboxylic acid cycle, and carotenoid biosynthesis were shown to be differentially regulated during fruit development and ripening of the sweet orange variety ‘Anliu’ and its red-fleshed mutant ‘Hong Anliu’ [10]. Metabolic shifts occurred in the green-white-red stages of strawberry that were associated with differential gene expression, and it was found that oxidative phosphorylation plays an important role in the regulation of fruit maturation [11].

Whole-genome sequencing of the PN40024 genotype of grapevine, originally derived from Pinot Noir, was completed in 2007 and has provided a useful resource for functional genomic studies [12]. A transcriptome analysis revealed that reduced biosynthesis, photosynthesis, and transport was the main reason for delayed senescence of the peel [13]. Most genes showed comparable expression levels between ‘Kyoho’ berry and its early-ripening mutant ‘Fengzao’ [14], and an analysis of differentially expressed genes (DEGs) revealed that those related to oxidative stress genes likely promote the early ripening of ‘Fengzao’ grape berry. Genes involved in carbohydrate metabolism and regulation of flavonoid metabolism and those of the solute carrier family showed the most marked changes in expression in ‘Kyoho’ and transgenic berry peels [15], and it was later reported that *V. vinifera VACUOLAR H*^*+*^*-PPASE 1* was activated by the MYB transcription factor MYBA1 and that hexokinase-mediated glucose signaling increased the expression of anthocyanin biosynthesis and transport-related genes to promote anthocyanin accumulation in grape peel. In addition, differences in the levels of microRNAs (miR169-NF-Y subunit, miR398-CSD, miR3626-RNA helicase, miR399-phosphate transporter, and miR477-GRAS transcription factor) and their targets have been observed in ‘Kyoho’ and ‘Fengzao’ during berry development and ripening [16].

DNA methylation is a mitotically reversible and meiotically heritable epigenetic modification [17] that is important in plant growth and development [18–20]. Recent studies have shown that DNA methylation is associated with fruit development and ripening [21–26]. Treatment with 5-azacytidine (5-azaC), a methyltransferase inhibitor, was shown to affect the development of tomato [27], strawberry [28], and *Acca sellowiana* [29] fruit by decreasing DNA methylation levels, resulting in an early ripening phenotype. Although 5-azaC treatment delayed fruit ripening in sweet orange [30], it also had a genome-wide demethylating effect [31]. 5-AzaC promoted the early ripening of grape berry and reduced global methylation level at a concentration of 100 μΜ in our previous study [32]. However, the mechanism by which 5-azaC alters gene expressions to accelerate berry ripening remains unknown.

To answer this question, in this study we carried out RNA-sequencing (RNA-seq) analysis of ‘Kyoho’ grape berry at five different stages of fruit development after 5-azaC treatment. The results provide novel insight into the molecular basis of grape berry ripening and a basis for future molecular studies.

## Results

### Analysis of RNA-seq libraries

To identify the genes involved in grape berry development, we performed transcriptome sequencing of ‘Kyoho’ grape berry with or without 5-azaC treatment at different developmental stages. The RNA-seq data have been uploaded to the National Center for Biotechnology Information Sequence Read Archive under the accession number PRJNA542248. A total of 30 cDNA libraries were constructed comprising 1.37 billion raw reads; 1.33 billion clean reads (accounting for 96.74% of raw reads) were recorded after removing adapter sequences and reads of low quality and those with more than 5% N bases. The average number of clean reads per sample was about 45.76 million and the clean Q30 (sequencing error rate < 0.1%) base rate was > 93.6% for each sample. Ultimately, 1.21 billion high-quality reads (accounting for 91.32% of clean reads) were mapped to the grape reference genome; 29.56 million of these were mapped to multiple locations in the genome at a ratio of 2.23% (Additional file 1).

In the 5-azaC-treated and untreated control samples, more genes were expressed at the A3 (23883) stage than at the C3 (22710) stage, whereas fewer genes were expressed at the other four stages. We also analyzed the number of genes expressed at different levels (fragments per kilobase million [FPKM] ≥ 50, 50 > FPKM ≥10, 10 > FPKM ≥2, 2 > FPKM ≥0.1, FPKM < 0.1) and found that the number of genes with FPKM ≥10 was higher in berries at A2 and A3 stages than in berries at stages C2 and C3; the number of genes with different expression levels was greater at C2 than at A2 (Additional file 2).

### Gene expression profile following 5-azaC treatment

**Fig. 1:**
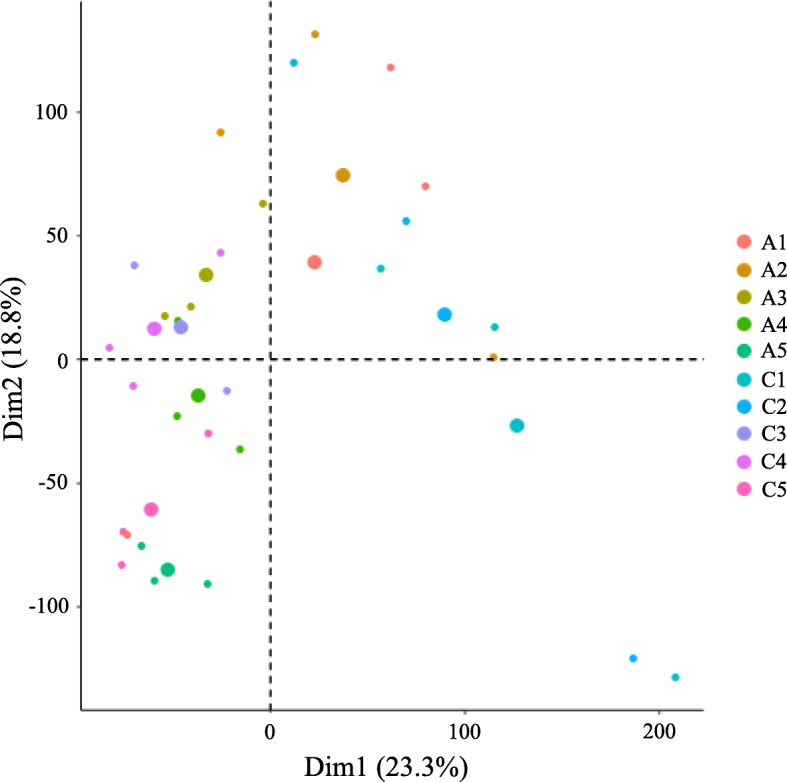
Principal component analysis of the RNA-Seq data. C and A represent the control and the treatment with 100 μM 5-azaC, respectively. The small icon indicates the original samples, the corresponding large icon of the same color and shape indicates the ‘center position’ of the group

Principal component analysis (PCA) revealed the heterogeneity of grape samples at different developmental stages based on gene expression in all samples. Dim1 and Dim2 had values of 23.3 and 18.8%, respectively, and accounted for 42.1% of the principal components (Fig. 1). PCA also revealed a consistency (i.e., no differences) between the three replicates at each developmental stage. Samples in the treatment and control groups at the same developmental stage clustered together, reflecting a lack of difference between them. On the other hand, samples at different developmental stages were dispersed irrespective of treatment condition, indicating that they differed significantly.

**Fig. 2:**
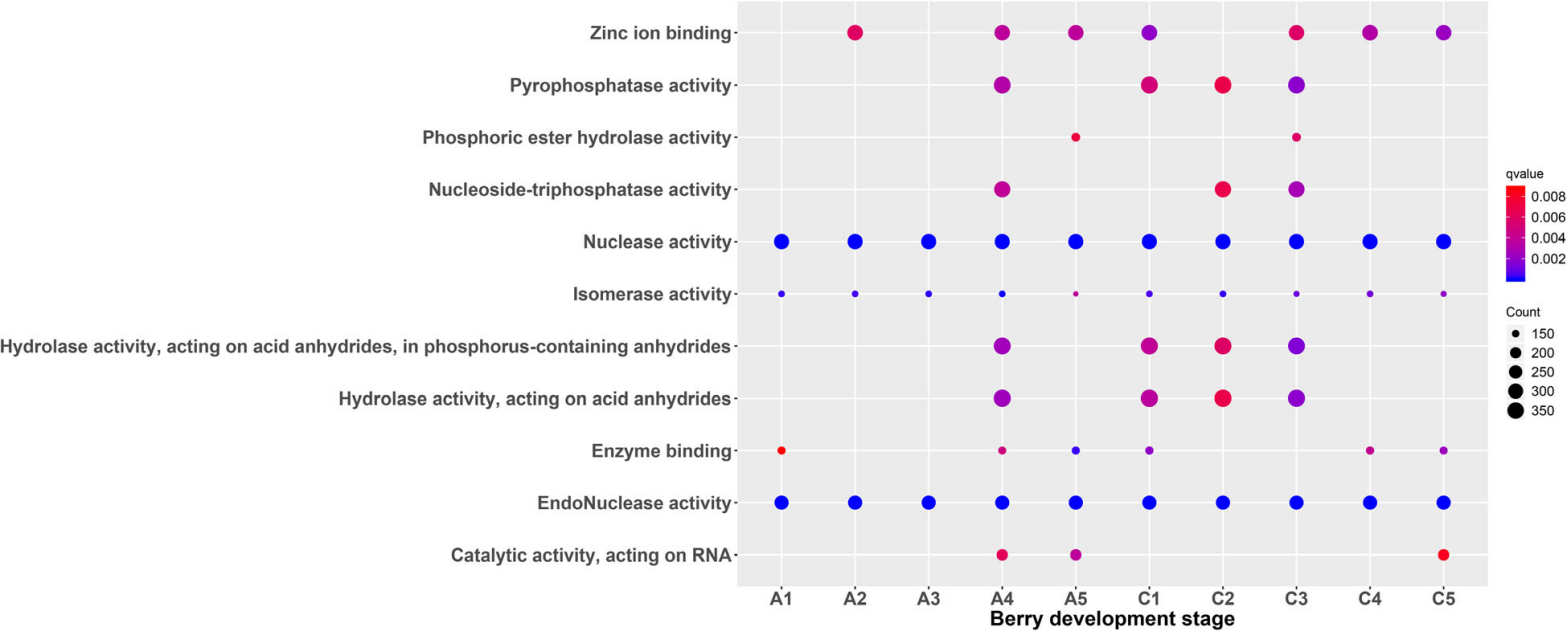
GO function enrichment analysis of gene expression at different developmental stages of grape berries. C and A represent the control and the treatment with 100 μM 5-azaC, respectively; the number of genes annotated in specific GO function is expressed as the size of the circle

We carried out a Gene Ontology (GO) enrichment analysis in order identify the biological processes in berry development that were affected by 5-azaC treatment and identified 11 enriched GO terms including those related to Zinc ion binding, Pyrophosphatase activity, Nucleoside triphosphatase activity, Nuclease activity, Hydrolase activity, and Endonuclease activity (Fig. 2). The number and significance of genes related to Nuclease activity, Endonuclease activity, and Isomerase activity were similar for control and treatment groups. Pyrophosphatase activity and Hydrolase activity (acting on acid anhydrides) were significantly enriched after 5-azaC treatment at stages C1, C2, and C3 (with the highest fold enrichment at C3) as well as at stage A4. Meanwhile, Nucleoside triphosphatase activity was enriched at C2, C3, and A4 (Fig. 2).

### Comparison of overall expression patterns by time course sequencing (TCseq) analysis

**Fig. 3:**
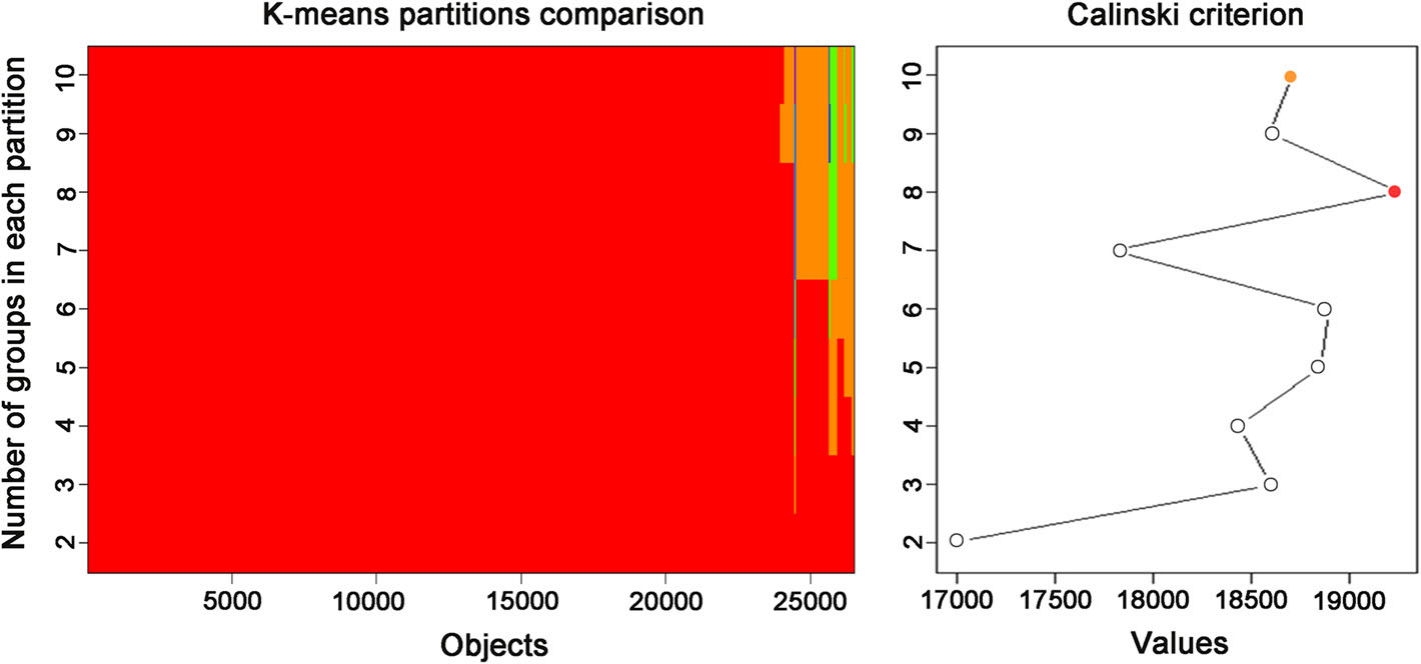
Grouping optimization of gene expression patterns for TC-seq analysis based on Calinski criterion value

**Fig. 4:**
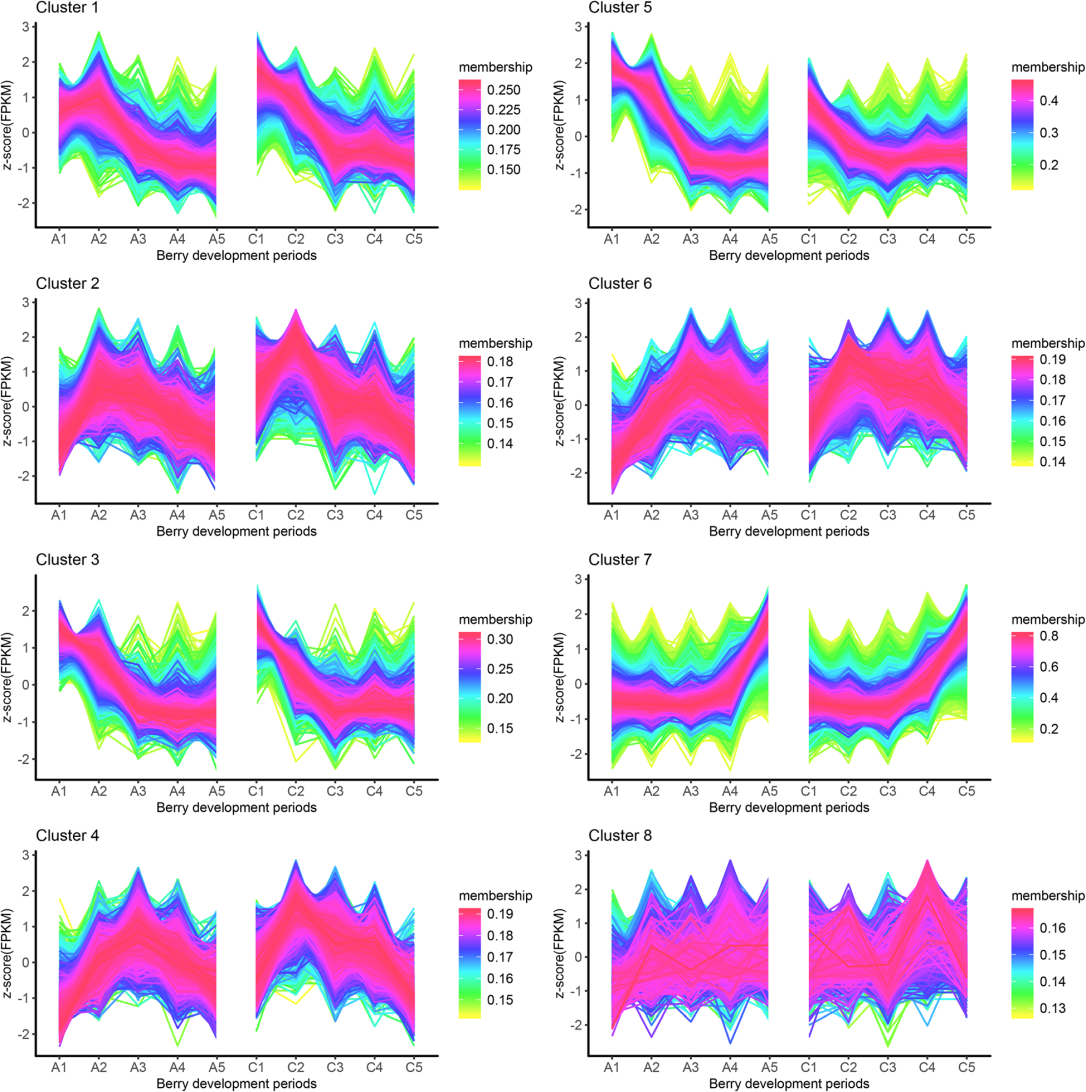
Cluster analysis of the gene expression patterns in the berry of the control and the treatment of ‘Kyoho’ across various developmental stages. Clustering was performed based on TCseq analysis and the number of genes included in each of the clusters is indicated on the top of the figure. The Y axis represents the FPKM values using 2 as the log base of a gene at different developmental stages. The X-axis represents the development stages of the berry. The gray lines indicate the change in gene expression level between samples. The dark purple lines represent the mean of the genes

To determine the overall expression patterns of genes common to the treatment and control groups, categories with different expression patterns were identified by TCseq analysis. Genes with similar expression patterns clustered together, with the highest Calinski criterion value occurring in eight clusters, suggesting that this was the optimal number of clusters (Fig. 3). The gene numbers for clusters 1–8 ranged from 1414 (cluster 8) to 6213 (cluster 5). Genes in each cluster showed very similar expression patterns overall in the treatment and control groups, whereas those in different clusters showed distinct expression patterns (Fig. 4).

**Fig. 5:**
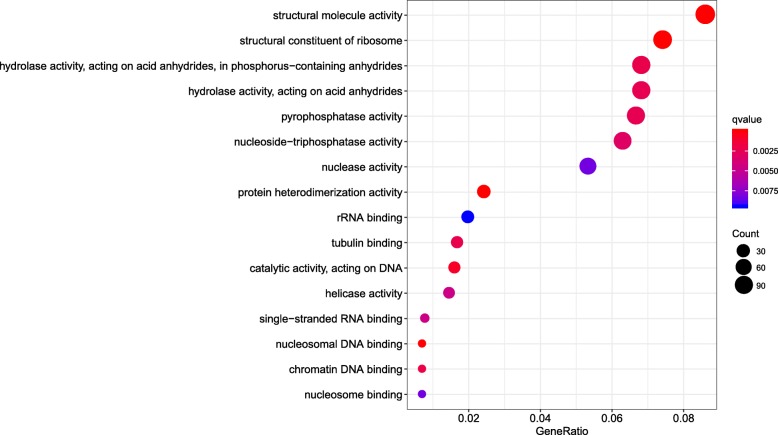
GO functional enrich analysis of genes in cluster 1 based on TCseq

The genes in clusters 1–8 showing similar expression patterns between treatment and control groups (Additional file 3) were divided into the following four classes. The expression of genes in cluster 2/4/6 first increased and then decreased with berry development; gene expression in cluster 3/5 gradually decreased before reaching a plateau; genes in cluster 7 showed relatively stable expression levels at the early stage of berry development followed by gradual upregulation; and the level of genes in cluster 8 remained constant across developmental stages. Cluster 1 comprising 4261 genes was exceptional; gene expression at C1 decreased gradually with berry development, but the level at A1 was lower than at C1. GO enrichment analysis of genes in cluster 1 revealed significant grouping of 16 GO terms (Fig. 5) including Structural molecule activity, Structural constituent of ribosome, Pyrophosphatase activity, Nucleoside triphosphatase activity, Hydrolase activity, Protein heterodimerization activity, and Tubulin binding (Additional file 4).

### Analysis of differentially expressed genes (DEGs)

We compared the transcriptional profiles of the treatment and control groups at the various stages of berry development and identified DEGs at each stage except for A1_C1. The number of DEGs between treatment and control groups at a given stage varied from 9 (A3_C3) to 690 (A5_C5). The expression of all 11 DEGs in A2_C2 was decreased (Table 1). The number of DEGs between successive developmental stages was 605 and 2188 for the control group and 104 and 2929 for the treatment group. There were fewer DEGs in A1_A2, A2_A3, and A3_A4 compared to C1_C2, C2_C3, and C3_C4, and the number of DEGs (up- and downregulated) was greater in A4_A5 than in C4_C5. The number of DEGs between successive developmental stages within treatment and control groups was greater than that between treatment and control groups at the same stage (Table 1).

**Table 1:** Numbers of DEGs in each developmental stage or in two adjacent stages of ‘Kyoho’ grape berry for control and 5-azaC treatment

Name	Up	Down	Total
A1_C1	0	0	0
A2_C2	0	11	11
A3_C3	7	2	9
A4_C4	10	9	19
A5_C5	261	429	690
A_C	1	2	3
C1_C2	1191	932	2123
C2_C3	1112	349	1461
C3_C4	181	424	605
C4_C5	1381	807	2188
A1_A2	31	73	104
A2_A3	509	495	1004
A3_A4	380	224	604
A4_A5	1862	1067	2929

DEGs were identified according to q < 0.05 and |log2FoldChange| ≥ 1

There were very few DEGs at the early stage of development; notably, no DEGs were identified in A1_C1. All DEGs (11) identified in A2_C2 were significantly downregulated after 5-azaC treatment. Functional annotation showed that these genes were Non-specific lipid-transfer protein A (VIT_12s0028g01180, LTA), Endochitinase (VIT_00s1290g00010), Purple acid phosphatase 15 (VIT_05s0029g00200, PAP15), Probable xyloglucan endotransglucosylase/hydrolase protein 23 (VIT_11s0052g01230, XTH25), and Basic 7S globulin (VIT_14s0128g00200). One of the genes, *XTH25*, is involved in the xyloglucosyl transferase pathway (Table 2).

**Table 2:** DEGs between the treatment and the control at the same developmental stage. (q < 0.05)

Groups	Gene ID	Log_2_FC	*P* value	q value	Annotation	KEGG pathway
A2_C2	VIT_13s0067g01240	−5.06	5.18E-07	0.01143	Protein EMB-1	–
	VIT_12s0028g03260	−8.26	1.95E-06	0.021544	Tyrosine aminotransferase	
	VIT_00s1290g00010	−7.20	4.04E-06	0.029672	Endochitinase	–
	VIT_05s0029g00200	−4.71	8.23E-06	0.030129	Purple acid phosphatase 15	–
	VIT_11s0052g01230	−6.63	7.97E-06	0.030129	Probable xyloglucan endotransglucosylase	xyloglucan: xyloglucosyl transferase
					/hydrolase protein 23	
	VIT_15s0048g02530	−4.64	9.56E-06	0.030129	NDR1/HIN1-like protein 12	–
	VIT_19s0015g00540	−5.83	5.65E-06	0.030129	–	–
	VIT_00s0450g00010	−8.23	1.26E-05	0.031395	S-linalool synthase	–
	VIT_12s0028g01180	−5.99	1.42E-05	0.031395	Non-specific lipid-transfer protein A	–
	VIT_14s0128g00200	−7.09	1.29E-05	0.031395	Basic 7S globulin	–
	VIT_09s0002g06070	−4.93	2.19E-05	0.04396	Late embryogenesis abundant protein Dc3	–
A3_C3	VIT_17s0000g04750	1.20	4.61E-08	0.000642	UDP-glycosyltransferase 89B2	–
	VIT_15s0048g00500	−1.28	9.44E-08	0.000658	Probable pectinesterase/pectinesterase inhibitor 36	–
	VIT_00s0301g00100	−1.24	8.84E-07	0.004107	Endonuclease 1	–
	VIT_05s0049g00770	3.26	3.39E-06	0.00946	–	–
	VIT_13s0064g01760	1.59	3.14E-06	0.00946	Beta-glucosidase 13	beta-glucosidase
	VIT_18s0001g11860	1.46	6.06E-06	0.012073	Probable galacturonosyltransferase-like 1	–
	VIT_04s0069g00860	2.26	1.11E-05	0.019415	Probable sarcosine oxidase	PIPOX; sarcosine oxidase/L- pipecolate oxidase oxidase1111111pipecolapipecolate oxidase pipecolate oxidase
	VIT_18s0117g00100	6.79	2.50E-05	0.031624	–	–
	VIT_07s0005g01380	2.03	4.40E-05	0.047143	Histidine kinase CKl1	–
A4_C4	VIT_13s0074g00700	−2.19	3.71E-11	5.45E-07	Pleiotropic drug resistance protein 2	–
	VIT_19s0014g00360	1.79	8.20E-10	3.24E-06	Protein ROOT INITIATION DEFECTIVE 3	–
	VIT_11s0016g02520	−1.14	8.29E-09	2.73E-05	–	–
	VIT_13s0067g02710	1.78	1.62E-08	4.58E-05	–	–
	VIT_01s0011g01300	1.96	2.13E-07	0.000468	Polygalacturonase QRT3	–
	VIT_19s0027g01820	2.30	1.27E-06	0.002514	Probable potassium transporter 13	–
	VIT_18s0001g08200	1.25	1.58E-06	0.002605	Protein DETOXIFICATION 48	TC.MATE, SLC47A, norM, mdtK, dinF; multidrug resistance protein, MATE family
						resistance protein, MATE family
	VIT_09s0002g00550	−1.46	2.24E-06	0.003152	GDSL esterase/lipase 1	–
	VIT_18s0001g06840	−3.49	2.14E-06	0.003152	Cationic peroxidase 1	–
	VIT_14s0006g02570	−1.91	2.46E-06	0.003237	Non-specific lipid-transfer protein 2	–
	VIT_04s0008g05730	1.79	4.34E-06	0.00476	Probable sucrose-phosphate synthase 1	–
	VIT_06s0004g04010	2.22	4.12E-06	0.00476	Exocyst complex component EXO70A1	EXOC7, EXO70; exocyst complex component 7
	VIT_07s0005g00030	−1.33	4.26E-06	0.00476	Glutathione S-transferase	–
	VIT_09s0002g08030	1.71	5.09E-06	0.005292	Arogenate dehydrogenase 2, chloroplastic	TYRAAT; arogenate dehydrogenase (NADP+), plant
	VIT_12s0028g00560	−4.23	7.27E-06	0.006841	–	–
	VIT_15s0046g01570	−4.55	7.19E-06	0.006841	Acidic endochitinase	–
	VIT_08s0007g01370	1.72	1.27E-05	0.010939	–	–
	VIT_18s0122g01430	1.96	2.72E-05	0.02063	Probable flavin-containing monooxygenase 1	FMO; dimethylaniline monooxygenase (N-oxide forming) forming)
						forming)
	VIT_16s0022g00510	−1.67	4.68E-05	0.032756	23.6 kDa heat shock protein, mitochondrial	HSP20; HSP20 family protein

Nine DEGs in A3_C3 were annotated; two of these—encoding Probable pectinesterase/pectinesterase inhibitor 36 (VIT_15s0048g00500, PE) and Endonuclease 1 (VIT_00s0301g00100)—were downregulated after 5-azaC treatment whereas seven genes encoding UDP-glycosyltransferase 89B2 (VIT_17s0000g04750, UGT89B2), Probable galacturonosyltransferase-like 1 (VIT_18s0001g11860, GTL), Histidine kinase CKl1 (VIT_07s0005g01380), Beta-glucosidase 13 (VIT_13s0064g01760, BGLU13), and Probable sarcosine oxidase (VIT_04s0069g00860) were upregulated. *BGLU13* and VIT_04s0069g00860 are involved in the β-glucosidase and sarcosine oxidase/l-pipecolate oxidase pathways, respectively (Table 2).

A total of 19 DEGs were identified in A4_C4: Probable sucrose-phosphate synthase 1 (VIT_04s0008g05730), polygalacturonase (PG) QRT3 (VIT_01s0011g01300, QRT3), and Probable flavin-containing monooxygenase 1 (VIT_18s0122g01430) were upregulated; VIT_18s0122g01430, is involved in the flavin monooxygenase and dimethylaniline monooxygenase (NO-forming) pathways. Downregulated genes were Non-specific lipid-transfer protein 2 (VIT_14s0006g02570), Acidic endochitinase (VIT_15s0046g01570), Pleiotropic drug resistance protein 2 (VIT_13s0074g00700), GDSL esterase/lipase 1 (VIT_09s0002g00550), Cationic peroxidase 1 (VIT_18s0001g06840), Glutathione S-transferase (VIT_07s0005g00030), and 23.6-kDa heat shock protein (VIT_16s0022g00510, *HSP 23.6*). The *HSP23.6* gene belongs to the *HSP20* family (Table 2). A5_C5 had the most DEGs. The expression levels of 28 DEGs encoding heat shock proteins and belonging to *HSP20*, *HSP70*, *HSP90*, and HSF_DNA-binding gene families were downregulated after 5-azaC treatment, as were all 28 DEGs related to photosynthesis and some methyltransferase genes including VIT_04s0023g02290, VIT_05s0049g01650, VIT_12s0028g02370, and VIT_08s0007g08470 (Additional file 5).

We performed a Kyoto Encyclopedia of Genes and Genomes (KEGG) pathway analysis of the DEGs in the treatment and control groups at the same developmental stage and found that only DEGs in A5_C5 were significantly enriched in KEGG pathways—namely, Protein processing in endoplasmic reticulum, Photosynthesis, Photosynthesis antenna proteins, Galactose metabolism, Flavone and flavanol biosynthesis, Diterpenoid biosynthesis, and ABC transporters; most genes were involved in Protein processing in endoplasmic reticulum (Additional file 6) and DEGs related to Photosynthesis were downregulated. The expression patterns and details of representative genes in key pathways are shown in Additional file 5.

### Weighted gene correlation network analysis (WGCNA)

**Fig. 6:**
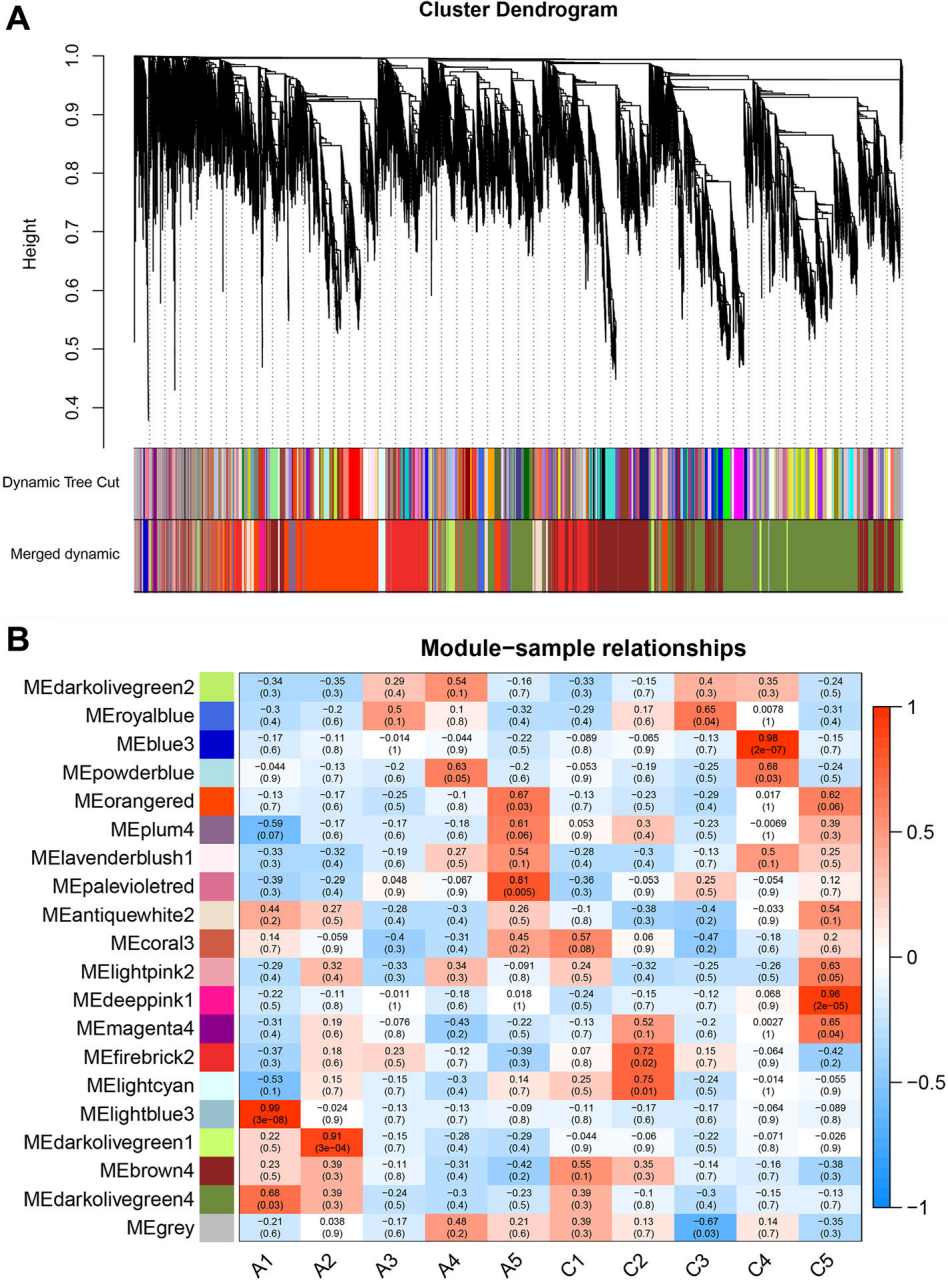
WGCNA of genes for control and treatment at each developmental stages from ‘Kyoho’ grape berry. **a** Hierarchical cluster tree shows coexpression modules identified by WGCNA. Each leaf represents one gene. The major tree branches constituted 20 modules labeled with different colors. **b** Module-sample association. Each row corresponds to a module, labeled with a color as in **a**. Modules are distinguished by different colors which were arbitrarily assigned by the WGCNA package. Each column corresponds to a developmental stage as indicated. The color of each cell at the row-column intersection indicates the correlation coefficient between the module and the developmental stage. A high degree of correlation between a specific module and the developmental is indicated in red

To gain insight into gene regulatory networks involved in the development of grape berry, we carried out a WGCNA of the transcriptome data of the five developmental stages of grape berry with or without 5-azaC treatment (19,387 genes, FPKM ≥0.5). In the initial module division, we set a soft threshold of 1 and used dynamic pruning to combine modules with a high similarity of characteristic genes (Fig. 6a). We obtained 20 gene modules with similar expression patterns; the total number of genes in each module ranged from 38 (palevioletred) to 6017 (darkolivegreen4). We analyzed the correlation between the characteristic genes of each module and berry development stage by calculating the Pearson correlation coefficient. The WGCNA results revealed that eight of the 20 modules were significantly correlated with a specific developmental stage (*P* < 0.05)—i.e., lightblue3, darkolivegreen1, powderblue, palevioletred, lightcyan, royalblue, blue3, and deeppink1 were significantly correlated with stages A1, A2, A4, A5, C2, C3, C4, and C5, respectively (Fig. 6b). Four of the modules were highly correlated with developmental stage (Pearson correlation coefficient > 0.9; *P* < 0.01), and the other modules were weakly correlated with five developmental stages in the treatment and control groups (Fig. 6b).

**Fig. 7:**
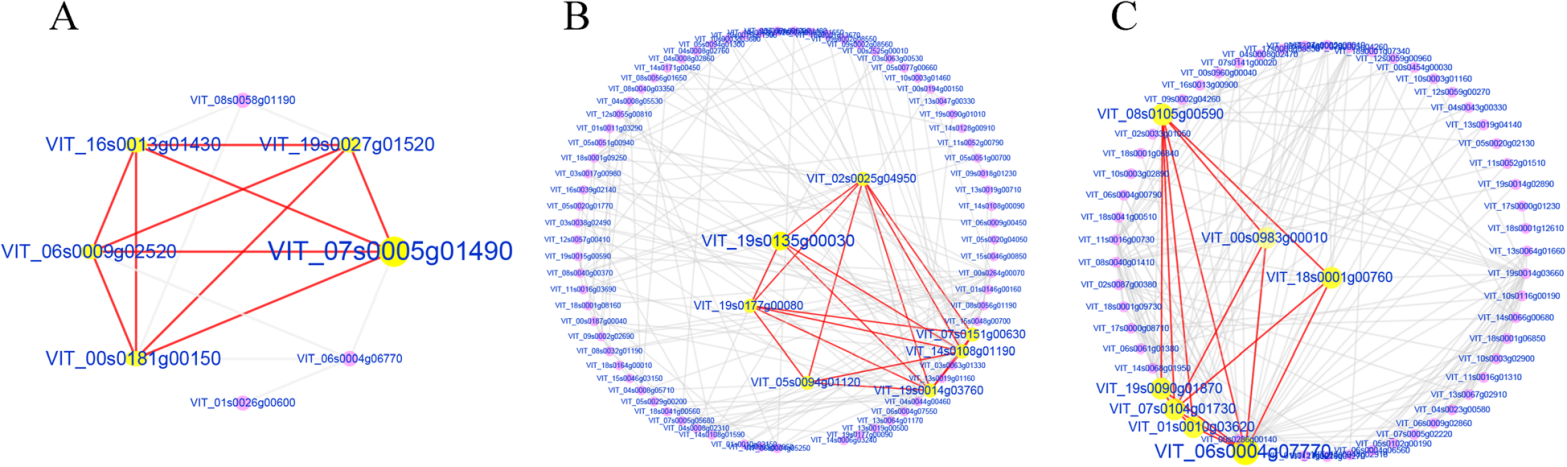
Correlation networks for the lightcyan, darkolivegreen1 and royalblue modules with C2, C3 and A2. Hub genes are indicated by yellow circles. **a** networks of C2; **b** networks of C3; **c** networks of A2

WGCNA can also be used to construct gene networks, where each node represents a gene and the connecting lines between genes represent co-expression correlations. Cytoscape v.3.6.1 was used to analyze the gene interaction network within the module and identify hub genes. The functional annotation revealed genes related to GDSL esterase/lipase, heat shock protein, caffeic acid 3-O-methyltransferase, and endoglucanase in the modules. We examined the gene regulatory networks in these modules and found that lightcyan was significantly correlated with stage C2 (r^2^ = 0.75), and included the VIT_07s0005g01490 (CLFSWN) gene encoding histone lysine N-methyltransferase EZA1 (Fig. 7a). Module royalblue, which was significantly correlated with C3 stage, included the VIT_19s0135g00030 (*OMT1*) gene encoding caffeic acid 3-O-methyltransferase (Fig. 7b). However, these two genes were not expressed after 5-azaC treatment. Module darkolivegreen1 was significantly correlated with A2 stage (r^2^ = 0.91) and included the hub gene VIT_06s0004g07770 encoding *POD4* (Fig. 7c).

### Validation by quantitative real-time (qRT-)PCR

**Fig. 8:**
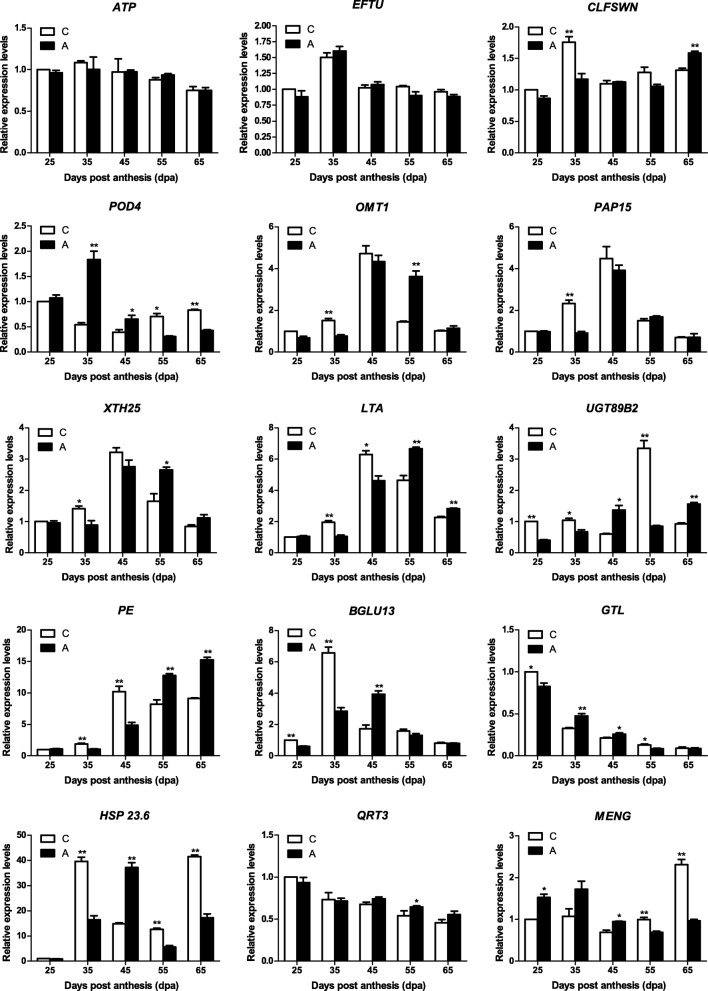
Expression profiles of selected genes from qRT-PCR in the 5-azaC treatment and the control. The x-axis represented different sampling date, while relative expression levels for the y-axis. Data represented the mean of three biological replicates. Error bars represented standard deviations from three independent technical replicates. The asterisks indicate the significant level (*P < 0.05, **P < 0.01) based on a Duncan’s multiple range test

We verified the accuracy and reproducibility of the results of the transcriptome analysis by qRT-PCR. We selected 15 genes—including nine that showed differential expression between the control and treatment groups at the same development stage, two (*ATP* and *EFTU*) from cluster 1 in the TCseq analysis, and three candidate hub genes (*CLFSWN*, *POD4*, and *OMT1*) identified in the WGCNA-for validation. *ATP* and *EFTU* showed similar expression levels in treated and control berries. The expression patterns of *CLFSWN*, *POD4*, and *OMT1* were similar to those obtained in the transcriptome analysis. The expression levels of *PAP15*, *XTH25*, and *LTA* were lower than those in the control group at 35 days post-anthesis (dpa); at 45 dpa, the levels of *UGT89B2*, *BGLU13*, and *GTL* after treatment were higher whereas that of *PE* was lower than in the control group. Treatment with 5-azaC decreased *HSP 23.6* expression at 55 dpa; *QRT3* expression was lower at 25 and 35 dpa but higher at 45 and 55 dpa than in the control group. *QRT3* expression during berry development was consistent with the transcriptome data, whereas the level of *MENG* in treated berries was lower than in the control group at 65 dpa. Overall, the relative expression of these genes during grape berry development as determined by qRT-PCR was consistent with and confirmed the accuracy and reliability of the transcriptome sequencing data (Fig. 8).

## Discussion

Tomatoes treated with 5-azaC ripen prematurely, and whole-genome bisulfite sequencing of the fruit at four stages of development has revealed many differentially methylated regions, suggesting that DNA methylation is an important regulator of fleshy fruit ripening [27]. As a DNA methyltransferase inhibitor, 5-azaC has been used to reduce methylation levels in other plants [28, 30, 33]. We previously showed that 5-azaC decreased methylation in developing ‘Kyoho’ grape berry [32]. 5-AzaC not only affects multiple physiological processes during plant development such as enhancing resistance and oxidation, inducing flowering, and influencing phenotype, but also affects gene expression during fruit maturation. RNA-seq is a useful tool for whole-genome expression profiling and identification of candidate genes related to this process. The TCseq results showed that the expression patterns of most genes were similar between the treatment and control groups (Fig. 4). No DEGs were identified in A1_C1 and only a few were detected in A2_C2, A3_C3, and A4_C4, confirming that 5-azaC treatment had little effect on the expression of most genes. However, there were 690 DEGs in A5_C5, which was more than the number in earlier developmental stages. Based on physiological indices and developmental state [32], 5-azaC was found to promote the early ripening of grape berry. Methylation normally leads to gene silencing but demethylation activates gene expression. 5-AzaC inhibited methylation but the DEGs expressed at A2 were downregulated, indicating that 5-azaC can also inhibit gene expression as previously reported [33]. Thus, 5-azaC treatment has pleiotropic effects on gene expression and related regulatory networks.

Fruit softening involves the dissolution of the cell wall due to polymer depolymerization [34]. The degradation of cell wall starch was shown to be the main cause of banana fruit softening/ripening [35, 36]. ‘Kyoho’ grape berry has a very hard texture at the very early stage of development, reaching peak hardness at 45 dpa before softening, which reflects the breakdown of the cell wall [32]. Our transcriptome analysis revealed that genes associated with cell wall softening such as *UGT89B2*, *GTL*, and *BGLU13* were significantly upregulated at 45 dpa after 5-azaC treatment (Table 1). Similarly, *QRT3* expression was increased at 55 dpa when the berries were at the softening stage, suggesting that it regulates berry ripening. Tomato genome sequencing revealed the expression of over 50 structural genes encoding known or putative cell wall-modifying proteins during fruit development and ripening [37]. Inhibiting PG activity was found to slow the softening of bayberry [38]. Phospholipase (PL) activity degrades cross-linked pectin polymers, and pectin polysaccharide in the cell wall is further degraded by PG, resulting in fruit softening; silencing PL expression slows the degree of fruit softening in strawberry [39]. As a PG, QRT3 was upregulated after 5-azaC treatment in early-ripening berries in this study. BG participates in the synthesis of abscisic acid, which is associated with grape ripening; *VvBG1* overexpression in strawberry increases BG activity and promotes fruit ripening [40]. Interestingly, we found that *BGLU13* was upregulated by 5-azaC treatment.

HSPs are responsible for protein folding, assembly, translocation, and degradation during normal cell growth and development [41]. HSP70, HSP90, and their co-chaperones are involved in signal transduction and protein targeting and degradation [42]. Small HSP is implicated in seed, pollen, and fruit development [43, 44]. *MiHSP17.6* expression, which regulates the development of mango fruit, declined during the early stage of fruit development, increased during the middle stage, and then decreased at the late stage; the highest expression level was at 60 dpa [45]. Class II *sHSP 17.4* mRNA was detected at all stages of the ripening process of tomato fruit and reached a maximal level at the later stage, whereas Class II *HSP 17.6* and *17.7* mRNA had the highest expression at the turning and pink stages, respectively [46]. *FaHSP17.4* expression was increased during cell division in strawberry fruit development, but gradually decreased during maturation after cell division ceased [47]. In this study, the *HSP 23.6* gene belonging to the HSP20 family was downregulated at 55 dpa following 5-azaC treatment, with other HSPs (e.g., *HSP20*, *HSP70*, and *HSP90*) showing a similar trend at 65 dpa. These results indicate that 5-azaC inhibits the expression of *HSP* genes although the underlying mechanism remains to be determined.

Protein phosphorylation plays an important role in the regulation of growth and development of strawberry fruit. Phosphorylated proteins not only participate in transcriptional regulation and cell division but also modulate the response to plant hormones and sugar metabolism, which are related to fruit ripening and softening [48]. In this study, GO enrichment analysis of genes at different developmental stages revealed factors that control berry development; their expression was stable and was not readily perturbed by exogenous substances. Moreover, they had related functions during berry development such as nuclease, isomerase, and endonuclease activities. However, the expression of some genes was altered at A4 by 5-azaC treatment including those encoding pyrophosphatase, nucleoside triphosphatase, and hydrolase, possibly as a result of changes in their methylation status. Meanwhile, the results of the TCseq analysis showed that most of the genes showed the same or similar expression patterns between treatment and control groups at different developmental stages, although gene expression patterns in cluster 1 were altered at 25 dpa. The GO enrichment analysis of cluster 1 genes also revealed that many were related to pyrophosphatase, nucleoside triphosphatase, and hydrolase activities.

PAP is produced by plant secretion under low phosphorus conditions and can hydrolyze phosphate groups on organophosphorus substrates and produce phosphorus for absorption and utilization by plants [49]. The PAP-related gene *MdPAP10* cloned from apple was found to be expressed in root, stem, leaf, flower and fruit, and its overexpression induced phosphorus-related gene expression [50]. In this study, 5-azaC treatment reduced the level of *PAP15* at 35 dpa, suggesting that this gene is related to grape berry development. However, the specific functions of PAP remain to be determined. In summary, our results suggest that grape berry ripening involves changes in protein phosphorylation state.

DNA methylation is generally considered to be a marker of transcriptional repression. However, decreased DNA methylation levels are also associated with the repression of genes, for instance during grape berry ripening; this is especially true in the case of genes related to photosynthesis, which are not required by ripened berries. Photosynthesis is important for the rapid growth of young fruit and starch production. With fruit ripening, the chloroplast differentiates into pigment cells and promotes coloration. Hypomethylation of DEGs is increased during chlorophyll biosynthesis. Accordingly, many photosynthesis-related genes were downregulated at A5_C5 (65 dpa). Whereas the berries of control plants were still green at this stage, those in the 5-azaC treatment group had changed color. Thus, 5-azaC may inhibit the expression of photosynthesis-related genes. However, additional studies are needed to clarify the molecular link between DNA methylation and gene expression changes during grape berry development and ripening.

## Conclusions

Transcriptome analysis of grape berry was performed at five developmental stages to elucidate the gene expression networks controlling ripening of ‘Kyoho’ grape berry following 5-azaC treatment. Most genes were detected at similar levels between the treatment and control groups in the time series analysis, indicating that 5-azaC treatment does not significantly influence the expression of most genes. Functional annotation of DEGs revealed that they were mainly related to fruit softening, photosynthesis, protein phosphorylation, and heat stress. The results provide insight into the mechanisms that regulate grape berry development, which is useful for establishing grape varieties with specific favorable characteristics.

## Methods

### Plant material and 5-azaC treatment

Plant samples were collected from 6-year-old hedgerow-cultivated ‘Kyoho’ grape vines growing in Yanshi County, Luoyang, China (34.41°N, 112.46°E) in 2017, and were identified and stored in our laboratory. Field management was conducted according to local agricultural practices. A total volume of 20 ml of the methyltransferase inhibitor—100 μM 5-azaC with Silwet-77 as the surfactant (0.3% v/v) (both from Solarbio, Beijing, China)—was evenly sprayed on each berry cluster while control plants were sprayed with water as previously described [32]. Two treatments were administered. Grape berries of uniform size and without mechanical damages were collected at five developmental stages-i.e., 25, 35, 45, 55, and 65 dpa. The berries were labeled as shown in Table 3 in order to facilitate subsequent analysis. The samples were immediately frozen in liquid nitrogen and stored at − 80 °C until further analysis.

**Table 3:** Sampling time points of ‘Kyoho’ grape berries for 5-azaC treatment and the control

	Sampling date (dpa)				
Control	C1 (25)	C2 (35)	C3 (45)	C4 (55)	C5 (65)
Treatment	A1 (25)	A2 (35)	A3 (45)	A4 (55)	A5 (65)

dpa-days post anthesis

### Library preparation and transcriptome sequencing

cDNA library preparation and transcriptome sequencing was performed as previously described [51]. Briefly, mRNA was enriched with oligo (dT) magnetic beads from total RNA. First-strand cDNA was synthesized using random hexamers with the fragmented mRNAs as templates; second strand buffer, dNTPs, RNaseH, and DNA polymerase I were then added to the reaction. The QIAQuick PCR kit and EB buffer were used for purification and elution, and end repair of the double-stranded cDNA was performed using A bases and an adapter. Fragments of the appropriate size were recovered by agarose gel electrophoresis and PCR amplification was performed to complete whole library preparation. RNA-seq libraries for the control and treatment groups were labeled as C and A, respectively. Three biological replicates of each library were sequenced on a HiSeq 2500 platform (Illumina, San Diego, CA, USA) with 150-bp paired-ends by Annoroad Gene Technology Co. (Beijing, China). RNA-seq data were uploaded to the Sequence Read Archive of the National Center for Biotechnology Information (accession number: PRJNA542248).

### RNA-seq data analysis

RNA-seq data analysis was performed according to previously published protocols [52]. To ensure data quality, clean reads were obtained by removing reads contaminated with adapters, those of low quality, and those in which N bases constituted > 5% of total bases. The Q30 of clean reads was calculated.

Bowtie2 v.2.2.3 was used to build a genome index and clean reads were aligned to the grape reference genome [12] using HISAT2 v.2.1.0 [53] with the BWT algorithm [54]. The expression levels of genes in each sample were calculated using HTSeq 0.6.0 and normalized, and are expressed as FPKM values to facilitate comparisons between different genes [52].

### Differential expression analysis

Linear regression was used to estimate gene expression levels in each sample and the probability value (*P* value) of each gene that was differentially expressed between the two groups was calculated with the Wald test. The P value was corrected with the BH method by multiple hypothesis testing to obtain the q value (q). DESeq2 v.1.6.3 [55] was used to analyze differential gene expression between the two groups. Genes with |log2[foldchange]| ≥ 1 and q < 0.05 in each pairwise comparison were identified as DEGs. The UniProt database was used to annotate and obtain detailed information on the DEGs.

### Cluster analysis

TCseq analysis was performed using the R packages Vegan and Cairo to evaluate trends in gene expression at different developmental stages of grape berry using FPKM values, and genes with similar expression patterns were divided into sets [56]; the gene expression trends in the same set were similar under the same conditions. To investigate gene function, GO and KEGG enrichment analyses were performed using R package (clusterProfiler). GO terms and KEGG pathways with q < 0.05 were considered as significantly enriched.

### WGCNA

To elucidate gene regulatory networks involved in grape berry development, WGCNA was performed for all genes (19,387) with FPKM ≥0.5 [57], with the soft threshold set to 1. WGCNA calculates the correlation between genes using a topological overlap method, which yields more biologically meaningful results. Cytoscape v.3.6.1 was used to analyze gene interaction networks in the module. Hub genes in a given module were screened with the MCODE module.

### Validation of DEGs by qRT-PCR

To validate the accuracy of RNA-seq data, the expression levels of some DEGs, genes of cluster 1 identified in the TCseq analysis, and candidate hub genes were evaluated by qRT-PCR. Total RNA was isolated from ‘Kyoho’ grape berries in the treatment and the control groups using the RNAprep Pure Plant Kit (Polysaccharides & Polyphenolics-rich) (DP441; Tiangen, Beijing, China) according to the manufacturer’s instructions. HiScript II 1st Strand cDNA Synthesis Kit (R211–01; Vazyme, Nanjing, China) was used for reverse transcription. Using a suitable amount of reverse transcribed cDNA as template, qRT-PCR amplification was performed on a CFX96 Real Time PCR Detection System (Bio-Rad, Hercules, CA, USA) using *TransStart* Top Green qRCP SuperMix (AQ131; Trans, Beijing, China) according to the manufacturer’s instructions. The reaction conditions were 95 °C (30 s), followed by 40 cycles at 95 °C (5 s) and 56 °C (30 s). The experiment was repeated three times and fold change in gene expression level was calculated with the 2^−ΔΔCt^ method [58]. The *VvUbiquitin1* gene was used as an internal reference. Primers used in this study are shown in Additional file 7. qRT-PCR data are presented as mean ± SD; the significance of differences between groups was evaluated with the independent samples t test.

## Supplementary information

**Additional file 1.** Summary of the read numbers aligned onto the grape reference genome.

**Additional file 2.** Summary of the gene expression numbers in the control and the treatment.

**Additional file 3.** The cluster information of the genes from TC-seq analysis.

**Additional file 4.** GO function enrichment analysis of gene expression in C1 cluster from TC-seq analysis.

**Additional file 5.** The information of DEGs for A5-C5.

**Additional file 6.** KEGG pathway analysis of DEGs for the treatment and the control at the same developmental stages.

**Additional file 7.** The sequences of primer used in the qRT-PCR.

## Data Availability

The RNA-Seq data supporting the results of this article have been uploaded to the Sequence Read Archive of NCBI (National Center for Biotechnology Information). It could be accessed via the NCBI SRA database with accession numbers of PRJNA542248 from 8th May 2020 onwards, as until then there is an embargo due to a complementary manuscript. Until then, these sequences are available from the corresponding author upon reasonable request.

## Abbreviations

5-azaC: 5-azacytidine
BG: β-glucosidases
BGLU13: Beta-glucosidase 13
CLFSWN: Histone-lysine N-methyltransferase EZA1
DEGs: Differentially expressed genes
dpa: Days post anthesis
FPKM: Fragments per kilobase per million mapped reads
GO: Gene Ontology
GTL: Probable galacturonosyltransferase-like 1
HSP: Heat shock protein
KEGG: Kyoto Encyclopedia of Genes and Genomes
LTA: Non-specific lipid-transfer protein A
MENG: Erythromycin 3″-O-methyltransferase
OMT1: Caffeic acid 3-O-methyltransferase
PAP15: Purple acid phosphatase 15
PG: Polygalacturonase
QRT3: Polygalacturonase QRT3
qRT-PCR: Real-Time Quantitative PCR
UGT89B2: UDP-glycosyltransferase 89B2
WGCNA: Weighted Gene Co-Expression Network Analysis
XTH25: Probable xyloglucan endotransglucosylase/hydrolase protein 23
