# Supplementary figures and images for: Transcriptome profiling of ‘Kyoho’ grape at different stages of berry development following 5-azaC treatment
Source: BMC Genomics. 2019 Nov 8;20:825. doi: 10.1186/s12864-019-6204-1 (PMC6839162; doi:10.1186/s12864-019-6204-1)

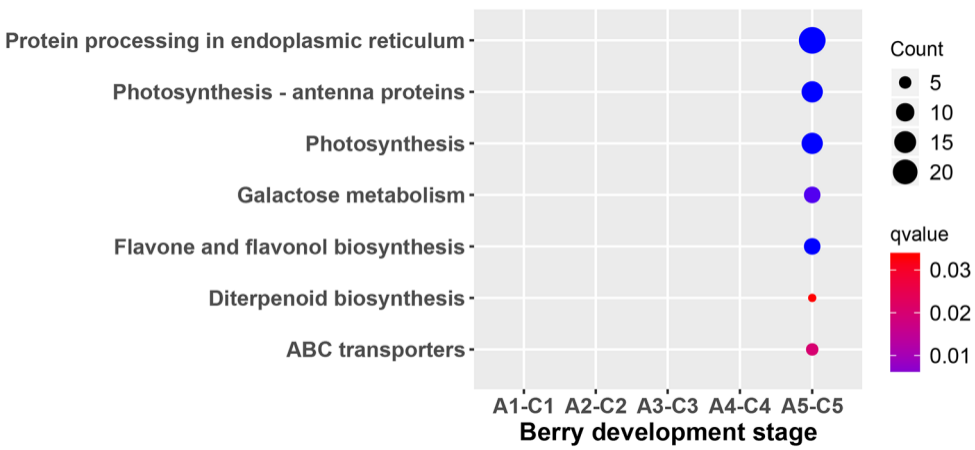

Supplement: Supplementary file 6 — Additional file 6. KEGG pathway analysis of DEGs for the treatment and the control at the same developmental stages. [file 12864_2019_6204_MOESM6_ESM.pdf]
